# Comparing Surgical and Oncological Outcomes between Indocyanine Green (ICG) Sentinel Lymph Node Mapping with Routine Lymphadenectomy in the Surgical Staging of Early-Stage Endometrioid Endometrial Cancer

**DOI:** 10.1155/2023/9949604

**Published:** 2023-10-17

**Authors:** Krystal Miao Lin Koh, Zheng Yuan Ng, Felicia Hui Xian Chin, Wai Loong Wong, Junjie Wang, Yong Kuei Lim

**Affiliations:** ^1^Department of Obstetrics and Gynaecology, KK Women's and Children's Hospital, 100 Bukit Timah Road 229899, Singapore; ^2^Department of Gynaecological Oncology, KK Women's and Children's Hospital, 100 Bukit Timah Road 229899, Singapore

## Abstract

**Methods:**

A retrospective single-centre cohort study of patients with early-stage endometrioid endometrial cancer undergoing staging surgery (total hysterectomy, bilateral salpingo-oophorectomy with/without pelvic lymph node, and/or para-aortic lymph node dissection (PLND)) with either SLN mapping or routine lymphadenectomy between July 2017 and December 2018.

**Results:**

203 cases with clinical and radiological International Federation of Gynaecology and Obstetrics (FIGO) stage I endometrioid endometrial cancer were included, out of which 109 cases underwent SLN mapping and 94 cases complete lymphadenectomy. Compared to the PLND group, the SLN group had shorter operative time (129 vs. 162 minutes), less blood loss (100 vs. 300 ml), and decreased length of postoperative hospital stay (3 vs. 4 days) (*p* < 0.001). The lymph node metastases detection rate was 4.6% and 7.4% for the SLN and PLND groups, respectively (*p* = 0.389). With a median follow-up of 14 months for the SLN and 15 months for the PLND group, the disease-free (DFS) and overall survival (OS) were comparable for both at 13 months (*p* = 0.538 and *p* = 0.333, respectively).

**Conclusion:**

SLN mapping has been shown to be an acceptable alternative to routine lymphadenectomy in the surgical staging of early-stage endometrial cancer in our centre, with a comparable lymph node metastases detection rate, DFS and OS, and reduction in operative morbidity. Our results with SLN mapping reproduce comparable outcomes to those reported in the literature.

## 1. Introduction

Endometrial cancer is the second most common gynaecological malignancy worldwide behind cervical cancer [1]. Complete lymphadenectomy has traditionally been considered the standard of care for assessing the lymph node status in patients with endometrial cancer. Though the therapeutic role of lymphadenectomy is questionable, especially in the early-stage uterine-confined endometrial cancer, its role in surgical staging affects the choice of adjuvant treatment [2, 3].

In recent years, there has been growing interest in the use of sentinel lymph node (SLN) mapping as an alternative to conventional lymphadenectomy to reduce the surgical morbidity associated with lymphadenectomy, namely, long term complications of lymphedema and lymphocyst formation. SLN mapping with ultrastaging has also been shown to increase the detection of lymph node metastases and improve staging and hence guiding adjuvant therapy [4, 5]. Indocyanine green (ICG) has been identified as the tracer of choice in may studies for SLN mapping in view of its superior detection rate compared to methylene blue and radiocolloid [6].

A SLN biopsy based on the Memorial Sloan Kettering SLN algorithm is now widely accepted as an alternative to a full lymphadenectomy in low-grade EC (grade 1 or 2 endometrioid), with numerous studies demonstrating its feasibility [6–9]. However, of yet there are few existing studies comparing the long-term surgical and oncological outcomes between ICG SLN mapping and routine lymphadenectomy for early-stage endometrial cancer.

In this study, we aim to compare the surgical and oncological outcomes between ICG sentinel lymph node mapping with routine lymphadenectomy in the surgical staging of early-stage endometrioid endometrial cancer within our centre.

## 2. Methods

This is a retrospective single-centre cohort study of patients with early-stage endometrioid endometrial cancer undergoing staging surgery (total hysterectomy, bilateral salpingo-oophorectomy (THBSO) with/without pelvic lymph node dissection (PLND), and/or para-aortic lymph node dissection (PAND)) with either ICG SLN mapping or routine lymphadenectomy between July 2017 and December 2018.

Inclusion criteria included all cases aged above 21 years, diagnosed with endometrioid endometrial cancer on pathology specimens from endometrial biopsy (outpatient endometrial sampling and dilatation and curettage specimens), clinically stage I disease (i.e., absence of extrauterine disease or regional/distal metastases based on physical examination findings and radiological findings with routine preoperative computed tomography (CT) and/or magnetic resonance imaging (MRI)), and medically fit to undergo surgery. Only endometrioid (type I) histology was included, including endometrioid histology with mucinous differentiation, and both laparoscopic and laparotomy approaches to surgery were included. Exclusion criteria included nonendometrioid histologies, clinical or radiological evidence of advanced cancer, synchronous ovarian and endometrial cancer, allergy to ICG dye or contraindications for receiving ICG tracer (e.g., liver failure), and lack of consent/capacity for consent or unwillingness to participate in the study.

For the ICG SLN mapping cases, we adopted the National Comprehensive Cancer Network (NCCN) SLN algorithm [6, 7, 10]. For each patient, a 25 mg vial of ICG powder (VERDYE(C), Diagnostic Green, AschheimDornach, Germany) was dissolved in 10 ml of sterile water (2.5 mg/ml) to obtain ICG dye. 1 ml of ICG dye was injected superficially (1–3 mm depth) and deep (1-2 cm depth) into the 3 and 9'o clock positions of the uterine cervix with a 22-gauge spinal needle. The intracervical ICG injection was performed either immediately after abdominal entry or after development of the retroperitoneal spaces. After opening the peritoneal spaces, the Karl Storz VITOM (R) ICG camera system with near-infrared spectroscopy (NIR) imaging was used for fluorescence detection of the lymph node channels and stations. The camera was either hand held or mounted on a stand depending on the surgeon preference. Identified SLNs were excised and sent for histological analysis. Lymph node removal was performed in accordance to the Memorial Sloan Kettering Cancer Center (MSKCC) surgical algorithm [11]. In each hemipelvis, if SLN mapping was not achieved, routine pelvic lymph node dissection was performed for that side. Complete pelvic lymph node dissection was then performed in the presence of intraoperative risk factors, such as extensive myometrial involvement on cut uterine specimen or suspicious lymph nodes. In preoperatively diagnosed high-grade endometrial cancer, routine pelvic and para-aortic lymph node dissection was performed.

The control group was patients who underwent THBSO with routine full lymphadenectomy (PLND with/without PAND). The ICG SLN mapping group included all cases that underwent ICG SLN mapping, including those who had contralateral unilateral lymphadenectomy or bilateral lymphadenectomy for unilateral mapping or no mapping, respectively, or those with full lymphadenectomy performed for clinically suspicious nodes intraoperatively.

Surgical outcome measures included operative time, estimated blood loss, and operative complications. Oncological outcomes defined were the number of cases upstaged, effect on adjuvant treatment, disease-free survival (DFS), and overall survival (OS).

Demographics and clinical data of the recruited patients and surgical data and histopathology data were extracted from a retrospective review of medical records and electronic database. Descriptive statistics were performed using SPSS (IBM, Armonk, NY).

The test of normality for the variables was performed using the Shapiro–Wilk test. For normally distributed variables, data are presented as mean and the 2 sample independent *t*-test used to compare the means. For nonnormally distributed variables, data are presented as median and the Mann–Whitney *U* test was used to compare medians. A *p* value less than 0.05 was taken as significant. For categorical variables, the chi-square test was used or Fisher's exact test if the expected count was less than 5. The Kaplan–Meier and log rank functions were used for DFS and OS.

## 3. Results

There were a total of 235 patients with clinical and radiological FIGO stage I endometrioid endometrial cancer cases from July 2017 to Dec 2018. After excluding 14 cases that did not meet the inclusion criteria, as well as 13 cases lost to follow-up and 5 cases with incomplete data, a total of 203 cases were included in the data analysis. This included 109 cases with ICG SLN mapping and 94 cases with systematic lymphadenectomy. 34% (*n* = 32) of the 94 cases undergoing systematic lymphadenectomy had para-aortic node dissection in addition to pelvic lymphadenectomy.

The mean age of the SLN mapping group was 55 -year-old compared to 59 -year-old in the routine lymphadenectomy (PLND) group (*p*=0.003). There was no significant difference in the median BMI (28.3 kg/m^2^ in the ICG group and 27.1 kg/m^2^ in the routine lymphadenectomy group, *p*=0.185). There was also no significant difference in the distribution of race (*p*=0.825) (Table 1).

The surgical outcomes of the two groups are summarized in Table 2. In terms of surgical outcomes, the median operative time, blood loss, and length of hospital stay postoperatively were significantly less in the SLN mapping group compared to the PLND group (*p* < 0.001). Median operative time was 129 minutes (range 62–251 minutes) in the SLN mapping group and 162 minutes (range 83–340 minutes) in the PLND group (*p* < 0.001). Median blood loss was 100 ml (range 10–500 ml) and 300 ml (10–1000 ml) in the SLN and PLND groups, respectively (*p* < 0.001). The median length of postoperative hospital stay was 3 days (range 1–43 days) for the SLN mapping group and 4 days (range 2–18 days) for the PLND group (*p* < 0.001). The one patient with a postoperative stay of 43 days in the SLN mapping group had a prolonged inpatient stay due to wound infection requiring secondary suture on the 21^st^ postoperative day.

There was no statistically significant difference in the number of operative complications between the two groups, with 14/109 (12.8%) of the patients in the SLN mapping group and 22/94 (23.4%) of patients in the PLND group (*p*=0.05) experiencing operative complications.

In the ICG group, the sentinel lymph node detection rate was 85.3% for bilateral mapping (93 cases, of which 3 showed both bilateral and para-aortic node mapping) and 10.0% for unilateral mapping. Only 4.6% of the cases had no mapping of any sentinel lymph nodes.

The median number of lymph nodes removed was 5 (range 0–34) in the SLN group and 20 (range 0–72) in the PLND group (*p* < 0.001). Despite the difference in the number of lymph nodes removed, the number of patients found to have lymph node metastases and number of patients who were upstaged were similar between the two groups (*p*=0.389 and *p*=0.395, respectively). 4.6% (5/109) of the patients in the SLN group had lymph node metastases, with 14.7% (16/109) patients being upstaged, compared to 7.4% (7/94) patients with metastases and 19.1% (18/94) in the PLND group being upstaged postoperatively.

There was no significant difference in the characteristics of the tumours in the SLN mapping and PLND groups in terms of the presence of lymphovascular space invasion (LVSI) (*p*=0.076), cervical stromal involvement (*p*=0.196), and positivity of peritoneal washings (*p*=0.08). In the SLN mapping group, 10/109 (9.2%) patients had LVSI, 8/109 (7.3%) had cervical stromal involvement, and 3/109 (2.8%) had positive peritoneal washings. This is similar to the PLND group in which patients LVSI, cervical stromal involvement, and positive peritoneal washings present in 19/94 (20.2%), 12/94(12.8%), and 9/94 (9.6%) patients, respectively.

The distribution of the final postoperative FIGO stage and grades of the patients for both the SLN mapping and PLND groups included are shown in Table 3.

There was no statistically significant difference between the preoperative tumour grade (from histological examination of in-office endometrial biopsy or endometrial curettings from dilatation and curettage) and the postoperative final histology between the two groups.

Preoperatively, in the SLN group, the distributions of grade 1, 2, and 3 tumours were 81.7%, 17.4%, and 0.9%, respectively, whereas in the lymphadenectomy group, the distribution was 44.7%, 36.2%, and 19.1%, respectively.

Postoperatively, the final histology showed the distributions of grade 1, 2, and 3 tumours to be 83.6%, 14.7%, and 1.8%, respectively, in the ICG group, and 46.8%, 39.4%, and 13.8%, respectively, in the lymphadenectomy group.

A significantly higher number of patients in the PLND group underwent adjuvant therapy compared to the SLN mapping group (60.6% in the PLND group compared to 26.6% in the SLN group, *p* < 0.001), with the majority undergoing radiotherapy as the mode of adjuvant therapy (51.1% and 20.2% in the PLND and SLN groups, respectively).

**Figure 1:**
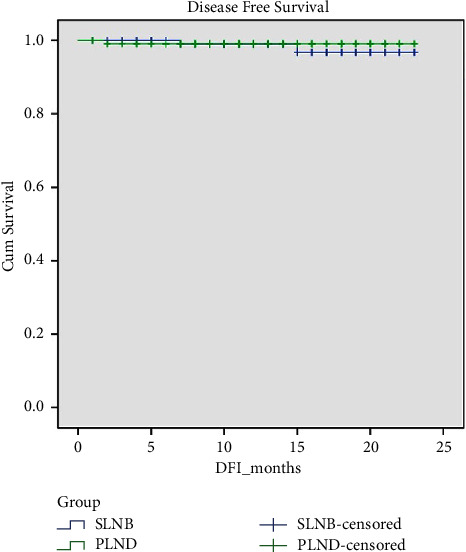
Comparing disease-free survival (DFS) between the SLN and PLND groups.

**Figure 2:**
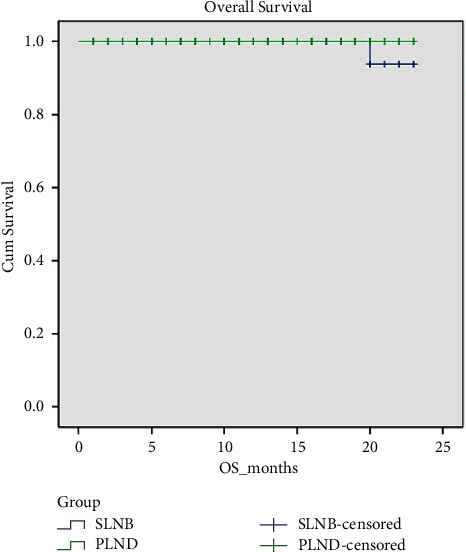
Comparing overall survival (OS) between the SLN and PLND groups.

There was no statistically significant difference in the number of recurrences, disease progression, or deaths between the ICG and PLND groups. With a median follow-up duration of 14 months (range 1–25 months) for the SLN group and 15 months (range 7–25 months) for the PLND group (*p*=0.177), the median DFS and OS for both groups was 13 months (range 1–23). This translates to a comparable DFS or OS between the two groups (*p*=0.538 and *p*=0.333, respectively), as demonstrated in Figures 1 and 2.

There was one recorded death in the ICG group. The patient was an 81 -year-old female with stage II grade 2 endometrioid cancer who underwent laparoscopic THBSO with ICG SLN mapping. The surgical procedure was uneventful and there were no operative complications. Her tumour was upstaged, but she declined adjuvant therapy. She developed recurrence in the pelvic lymph nodes and peritoneum 15 months postoperatively and eventually died 5 months later.

There were 2 patients who had disease recurrence in the SLN mapping group and this included the mortality case described above. The other patient had a stage IB grade 2 endometrioid adenocarcinoma who received adjuvant vault radiotherapy but still had a local recurrence in the vagina 7 months after treatment. In the PLND group, there was one recurrence and this was a patient who had a stage IB grade 2 endometrioid adenocarcinoma. She declined adjuvant radiotherapy and had a local recurrence in the vagina 2 months after surgery.

## 4. Discussion

The ASTEC trial in 2009 had shown no evidence of recurrence free or overall survival benefit with systematic pelvic lymphadenectomy for early stage endometrial cancer, with increased rates of lymphedema [3]. Despite criticisms of this trial for lacking a standardized lymphadenectomy protocol and inconsistencies in adjuvant therapy, clinical practice in many centers around the world changed as a result of this trial, with omission of systematic pelvic lymphadenectomy as a means of surgical staging for clinically early endometrial cancers.

However, the omission of lymphadenectomy for clinical early-stage cancers may potentially result in understaging some patients as the incidence of metastases to pelvic lymph nodes in patients with clinical stage I endometrial cancer who undergo systematic lymphadenectomy varies from 5% to 18% [12–15]. There are also limitations of preoperative selection for lymphadenectomy in early endometrial cancers. The accuracy of magnetic resonance imaging (MRI) extent of myometrial invasion and the presence of cervical and nodal metastasis has been shown to range from 66% to 90%. Underestimation of the FIGO stage occurs in 20%–30% of the patients and overestimation occurs in 13% of the cases [16–18]. Preoperative endometrial biopsy, regardless of sampling technique, including outpatient endometrial aspiration and dilatation and curettage additionally has limitations. Tumour type accuracy ranges from 74% to 92% and tumour grade accuracy ranges from 44% to 94% compared with final pathology results [19].

SLN mapping and biopsy have thus been suggested as an alternative surgical staging modality to bridge the gap between having no surgical staging whilst reducing the postoperative morbidity of a full lymphadenectomy procedure.

As described above, SLN biopsy based on the Memorial Sloan Kettering SLN algorithm is now widely accepted as an alternative to a full lymphadenectomy in low-grade EC (grade 1 or 2 endometrioid) and is recommended in the NCCN guideline [7]. The European Society of Gynaecological Oncology (ESGO) guideline also supports the consideration of SLN biopsy for staging in patients with low or intermediate risk disease and does not recommend systematic lymphadenectomy [20].

For surgical outcomes, there have been studies showing that ICG SLN mapping results in decreased operative time [21–23] and decreased blood loss [24] compared to full lymphadenectomy. In our study, the median operative time and blood loss were also significantly less in the SLN mapping group compared to the PLND group (*p* < 0.001). Median operative time was 129 minutes (range 62–251 minutes) and median blood loss was 100 ml (range 10–500 ml) in the SLN group compared to 162 minutes (range 83–340 minutes) and 300 ml (10–1000 ml), respectively, in the PLND group (*p* < 0.001). Although our results show no statistically significant difference in the number of operative complications between the two groups (14/109 (12.8%) of the patients in the SLN mapping group and 22/94 (23.4%) of patients in the PLND group, *p*=0.05), this may have been limited by the small sample size.

For oncological outcomes, in a 16–32 month follow-up duration, there were no reported differences in progression-free survival [25, 26]. Studies have found that ICG SLN mapping also increases the number of cases upstaged and consequently receiving adjuvant treatment [5, 21, 25, 27], which in turn has been shown to improve survival [28]. Our study similarly found that despite the difference in number of lymph nodes removed, the number of patients found to have lymph node metastases and number of patients who were upstaged were similar between the SLN and PLND groups (*p*=0.389 and *p*=0.395, respectively). 4.6% (5/109) of the patients in the SLN group had lymph node metastases, with 14.7% (16/109) patients being upstaged, compared to 7.4% (7/94) patients with metastases and 19.1% (18/94) in the PLND group being upstaged postoperatively.

Our study found comparable surgical and oncological outcomes between SLN mapping and routine lymphadenectomy. A few other studies have shown similar results. A meta-analysis of 3536 patients in 2019 showed that the recurrence rate was 4.3% and 7.3% after SLN biopsy and lymphadenectomy (*p*=0.63). Nodal recurrences were also similar between the two groups (1.2% vs. 1.7%; *p*=0.29) [29]. A retrospective study of 104 patients (52 patients undergoing SLN mapping and 52 patients undergoing lymphadenectomy) followed-up for 42 months showed a disease-free interval of 84.6% in the ICG group compared to 75.0% in the lymphadenectomy group (*p*=0.774) [30]. Another recent study of 360 patients (90 patients SLN mapping alone, 90 patients SLN mapping followed by lymphadenectomy, and a control group of 180 patients undergoing lymphadenectomy), with a median follow-up of 69 months showed no difference in disease-free survival (*p*=0.570) or overall survival (*p*=0.911) between the three groups [31]. Hence, SLN mapping appears to be noninferior to standard lymphadenectomy in terms of oncological outcomes.

In terms of SLN detection rates, the rates of SLN mapping in our study are comparable to a pilot study performed by Lim et al. on 35 patients undergoing laparoscopic SLN mapping, in which the overall detection rate of SLN was 97%, with 88.6% patients having bilateral mapping, 8.6% having unilateral mapping, and 2.9% having no mapping [10]. A retrospective study of 36 patients undergoing staging laparotomy for endometrial cancer also showed a similar overall SLN detection rate of 92% with bilateral mapping in 81% [32].

In terms of postoperative incidence of lymphedema, our study was not powered to compare lymphedema detection rates between the two groups. However, Leitao et al. showed that after controlling the use of adjuvant external beam radiotherapy and BMI, systemic lymphadenectomy had an increased prevalence of lower extremity lymphedema compared to SLN mapping (OR, 1.8; *p*=0.003). The study also found that patients with self-reported lymphedema had significantly worse quality of life compared to those without lymphedema [33]. Another study by Geppert et al. on 188 patients with endometrial cancer in Sweden who underwent robotic hysterectomy and ICG SLN biopsy similarly found that SLN biopsy resulted in a significantly lower incidence of lower limb lymphedema than complete lymphadenectomy (1.3% vs. 18.1%, *p*=0.0003) [34].

We recognize that our main limitation was the short median duration of follow-up of 14-15 months as well as the size of our study population, which may have affected the comparison of operative complications and long-term complications such as lymphedema. Longer term follow-up and the inclusion of a larger population of patients would be suggested for future studies.

Looking forward, the current focus of research is shifting to the use of SLN mapping in high grade endometrial cancers. The recent SENTOR study in 2020 found that SLN mapping is a feasible option for the surgical staging of high risk endometrial cancer [35]. Their study found that SLN mapping had a sensitivity of 96%, false-negative rate of 4%, and a negative predictive value of 99% for detection of nodal metastases in patients with high-grade endometrial cancer at an increased risk of nodal metastases and in fact improved the detection of node-positive cases compared with lymphadenectomy [35]. A more recent systematic review by Marchocki et al. similarly demonstrated that SLN biopsy can accurately detect lymph node metastases in high-grade endometrial cancer, with a pooled SLN detection rate of 91% per patient and 64% bilaterally and a pooled sensitivity and negative predictive value of 92% and 97%, respectively [36]. These findings suggest that SLN biopsy can potentially also replace routine lymphadenectomy in high-grade endometrial cancers.

## 5. Conclusion

In conclusion, our study findings suggest that ICG SLN mapping is an acceptable and safe alternative to routine lymphadenectomy in the surgical staging of early-stage endometrioid endometrial cancer in our centre, with a comparable lymph node metastases detection rate, disease-free survival and overall survival, and reduction in operative morbidity. Our centre's experience and results with SLN mapping successfully reproduce comparable surgical and oncological outcomes to those reported in the literature.

## Figures and Tables

**Table 1:** Demographics and perioperative findings of patients in the SLN mapping and PLND groups.

	Mean (range)		
	SLN^†^	PLND^‡^	*p* value (Mann–Whitney)^*∗*^
Age (years)	55 (31–85)	59 (30–79)	0.003

	Median (range)		
	SLN	PLND	*p* value^*∗*^
BMI (kg/m^2^)	28.3 (14.4–54.0)	27.1 (16.0–50.0)	0.185

	Number (%)		
	SLN (*n* = 109)	PLND (*n* = 94)	*p* value^*∗∗*^
Race			0.825

Chinese	76 (69.7%)	68 (72.3%)	

Malay	17 (15.6%)	16 (17.0%)	

Indian	6 (5.5%)	3 (3.2%)	

Others	10 (9.2%)	7 (7.4%)	

	Median (range)		*p* value^*∗*^
Median number of lymph nodes removed	5 (0–34)	20 (0–72)	<0.001

	Number (%)		
	SLN (*n* = 109)	PLND (*n* = 94)	*p* value^*∗∗∗*^
Number of patients with lymph node metastases	5 (4.6%)	7 (7.4%)	0.389

Number of patients upstaged	16 (14.7%)	18 (19.1%)	0.395

^†^SLN = sentinel lymph node mapping group. ^‡^PLND = pelvic lymph node dissection group. ^*∗*^Mann–Whitney *U* analysis for *p* value. ^*∗∗*^Chi-square analysis for *p* value. ^*∗∗∗*^Fisher's exact test for *p* value (count <5).

**Table 2:** Surgical outcomes between SLN mapping and PLND groups.

	Median (range)		
	SLN^†^	PLND^‡^	*p* value^*∗*^
Operative time (minutes)	129 (62–251)	162 (83–340)	<0.001
Blood loss (milliliters)	100 (10–500)	300 (10–1000)	<0.001
Hospital stay after surgery (days)	3 (1–43)^*∗*^	4 (2–18)	<0.001

	Number (%)		
	SLN^†^ (*n* = 109)	PLND^‡^ (*n* = 94)	*p* value^*∗∗*^

Number of operative complications	14 (12.8%)	22 (23.4%)	0.05

^†^SLN = sentinel lymph node mapping group. ^‡^PLND = pelvic lymph node dissection group. ^*∗*^Mann–Whitney for *p* value. ^*∗∗*^Chi-square analysis for *p* value.

**Table 3:** Postoperative pathological findings and the FIGO stage distribution in SLN mapping and PLND groups.

	Number (%)		
	SLN^†^ (*n* = 109)	PLND^‡^ (*n* = 94)	*p* value^*∗*^
Histological grade			
Grade 1	91 (83.6%)	44 (46.8%)	<0.001
Grade 2	16 (14.7%)	37 (39.4%)	
Grade 3	2 (1.8%)	13 (13.8%)	

Cervical involvement	10 (9.2%)	19 (20.2%)	0.076
Lymphovascular space invasion (LVSI) present	8 (7.3%)	12 (12.8%)	0.196
Peritoneal washings positive	3 (2.8%)	9 (9.6%)	0.08

Final FIGO stage (2009)	SLN (*n* = 109)	PLND (*n* = 94)	
1A	81 (74.3%)	50 (53.2%)	0.013
1B	14 (12.8%)	26 (27.7%)	
2	5 (4.6%)	10 (10.6%)	
3A	4 (3.7%)	1 (1.1%)	
3C1	5 (4.6%)	6 (6.4%)	
3C2	0 (0.0%)	1 (1.1%)	

^†^SLN = sentinel lymph node mapping group. ^‡^PLND = pelvic lymph node dissection group. ^*∗*^Chi-square analysis for *p* value.

## Data Availability

The data used to support the findings of this study are available on request from the corresponding author.
